# A non-linear connection between the total cholesterol to high-density lipoprotein cholesterol ratio and stroke risk: a retrospective cohort study from the China Health and Retirement Longitudinal Study

**DOI:** 10.1186/s40001-024-01769-9

**Published:** 2024-03-16

**Authors:** Binhui Xiao, Changchun Cao, Yong Han, Fangju Yang, Haofei Hu, Jiao Luo

**Affiliations:** 1 Department of Neurosurgery, Shenzhen Yantian District People’s Hospital, Southern University of Science and Technology Yantian Hospital, Shenzhen, 518081 Guangdong China; 2 Department of Rehabilitation, Shenzhen Second People’s Hospital, Shenzhen Dapeng New District Nan’ao People’s Hospital, No. 6, Renmin Road, Dapeng New District, Shenzhen, 518000 Guangdong China; 3 Department of Emergency, The First Affiliated Hospital of Shenzhen University, Shenzhen Second People’s Hospital, Shenzhen, 518035 Guangdong China; 4 Department of Nephrology, The First Affiliated Hospital of Shenzhen University, Shenzhen Second People’s Hospital, No.3002, Sungang West Road, Futian District, Shenzhen, 518035 Guangdong China

**Keywords:** Nonlinearity, Total cholesterol, High-density lipoprotein cholesterol, Total cholesterol to high-density lipoprotein cholesterol ratio, Stroke

## Abstract

**Objective:**

The connection between total cholesterol to high-density lipoprotein cholesterol (TC/HDL-C) ratio and stroke risk is controversial. This study aims to examine the connection between the TC/HDL-C ratio and stroke in middle-aged and older individuals who are part of the China Health and Retirement Longitudinal Study (CHARLS).

**Methods:**

This study conducted a retrospective cohort analysis, enrolling a total of 10,184 participants who met the designated criteria from CHARLS between 2011 and 2012. We then used the Cox proportional-hazards regression model to analyze the relationship between the TC/HDL-C ratio and stroke risk. Using a Cox proportional hazards regression model with cubic spline functions and smooth curve fitting, we were able to identify the non-linear relationship between the TC/HDL-C ratio and stroke occurrence. The sensitivity and subgroup analyses were also performed to investigate the connection between TC/HDL-C ratio and stroke.

**Results:**

This study revealed a statistically significant association between the TC/HDL-C ratio and stroke risk in subjects aged 45 years or older after adjusting for risk factors (HR: 1.05, 95%CI 1.00–1.10, P = 0.0410). Furthermore, a non-linear connection between the TC/HDL-C ratio and stroke risk was detected, with a TC/HDL-C ratio inflection point of 3.71. We identified a significant positive connection between the TC/HDL-C ratio and stroke risk, when the TC/HDL-C ratio was less than 3.71 (HR: 1.25, 95%CI 1.07–1.45, P = 0.0039). However, their connection was not significant when the TC/HDL-C ratio exceeded 3.71 (HR: 1.00, 95%CI 0.94–1.06, P = 0.9232). The sensitivity analysis and subgroup analyses revealed that our findings were well-robust.

**Conclusion:**

Our study demonstrated a positive, non-linear connection between the TC/HDL-C ratio and stroke risk in middle-aged and older individuals. There was a significant positive connection between the TC/HDL-C ratio and stroke risk, when the TC/HDL-C ratio was less than 3.71. The current research can be used as a guideline to support clinician consultation and optimize stroke prevention measures for middle-aged and older adults.

**Supplementary Information:**

The online version contains supplementary material available at 10.1186/s40001-024-01769-9.

## Background

Stroke is distinguished by its significant morbidity, disability, and mortality rates. As of 2019, stroke persisted as the third most prevalent cause of disability and the second most prevalent cause of death globally [1]. Moreover, the economic burden of stroke is substantial, resulting in significant healthcare expenditures and imposing a significant global burden [2].

The total cholesterol to high-density lipoprotein cholesterol (TC/HDL-C) ratio is a widely recognized lipid index that serves as a surrogate marker for the assessment of atherogenic dyslipidemia [3]. It reflects the balance between the cholesterol carried in atherogenic lipoproteins and that in anti-atherogenic high-density lipoprotein particles [3]. A higher TC/HDL-C ratio indicates a higher proportion of cholesterol in potentially harmful lipoproteins relative to protective high-density lipoprotein, and it has been correlated with an increased risk for atherosclerotic cardiovascular diseases, including stroke. The current research landscape presents a body of evidence supporting the TC/HDL-C ratio as a predictor of diabetes, prediabetes, chronic kidney disease and cardiovascular events [4]. Existing evidence underscores an association between an elevated TC/HDL-C ratio and an augmented risk of stroke [5–7], although contradictory findings have been reported [8–10]. A cohort study conducted in Paris contradicts the prevailing notion, asserting that the TC/HDL-C ratio does not exhibit a significant association with ischemic stroke [10]. Similarly, a prospective investigation involving 24,566 adults, with an average follow-up period of 2.7 years, fails to establish a substantial correlation between the TC/HDL-C ratio and the risk of stroke [9]. Controversial results may be due to the short follow-up period of previous studies, limited adjustment for confounding variables, and the fact that these studies were all based on linear regression models exploring the relationship between TC/HDL-C ratio and stroke. However, it is known that high levels of TC and low levels of HDL-C are considered risk factors for stroke. Therefore, we propose a hypothesis that elevated TC/HDL-C ratio may still be a risk factor for stroke. In addition, taking into account the difference in the distribution range of the TC/HDL-C ratio, there may be a nonlinear relationship between TC/HDL-C ratio and stroke. Therefore, this study aims to elucidate the relationship between the TC/HDL-C ratio and the risk of stroke in middle-aged and older adults participating in the China Health and Retirement Longitudinal Study (CHARLS).

## Methods

### Data source

This cohort study utilized data from the CHARLS which is a nationally representative cohort study spanning the years 2011 to 2018. The CHARLS sample was obtained from 450 communities in 150 districts and 28 provinces through multi-stage probability sampling, with 10,257 households participating in the baseline survey and 17,708 participants. Further comprehensive information regarding the CHARLS can be found elsewhere [11]. In summary, the CHARLS employs a face-to-face interview methodology and a structured questionnaire to collect data from a nationally representative sample of the Chinese population. The primary focus of the survey is to gather information on health-related data, lifestyle-related data, and socio-demographic data. Additionally, the CHARLS incorporates a range of physical measurements and the collection of blood samples. The initial survey was carried out in 2011, and subsequent follow-ups were conducted every 2 years for all participants. Researchers interested in accessing and downloading this study's data and relevant information can do so through the official CHARLS project website (http://charls.pku.edu.cn/) [11].

### Study population

The Peking University Biomedical Ethics Review Board granted authorization for the implementation of the CHARLS investigation, and all participants duly provided written informed consent [11]. Furthermore, our research adhered to the principles outlined in the Declaration of Helsinki. All procedures were conducted in compliance with the requisite standards and legislation.

**Fig. 1:**
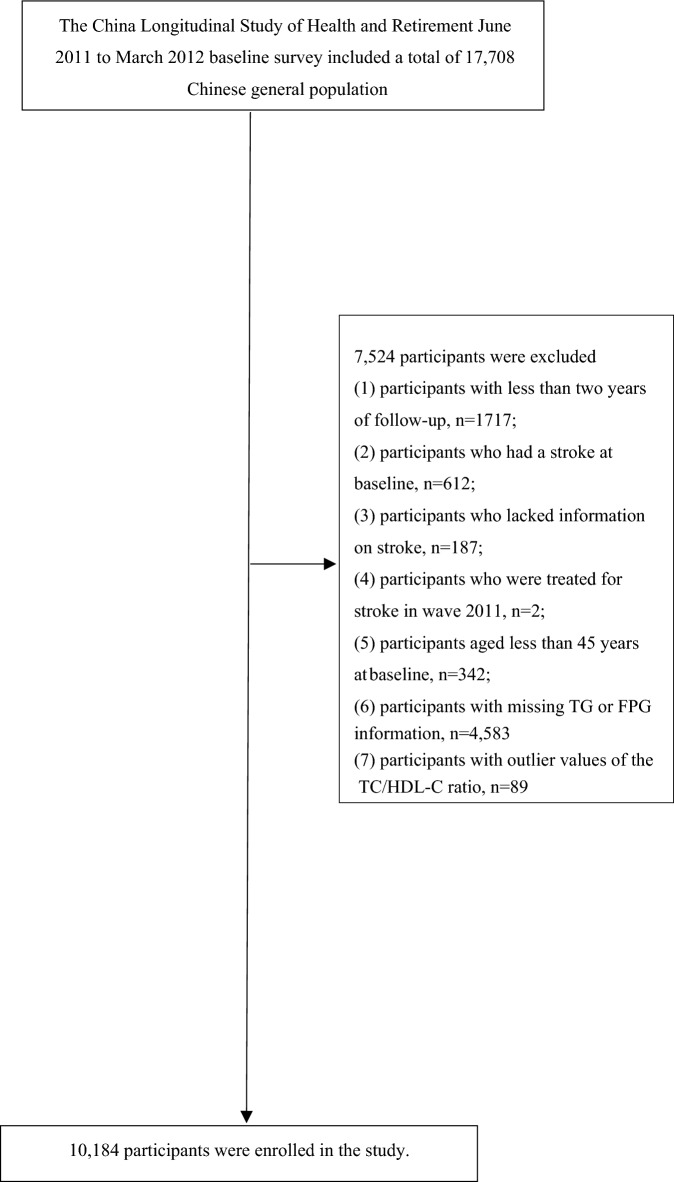
Study population

Our study utilized data from four waves of the CHARLS conducted in 2011, 2013, 2015, and 2018. We selected the baseline participants from the CHARLS 2011 dataset (n = 17,708). Of these, we excluded 7524 individuals from the analysis for the following reasons: (1) a follow-up period of less than 2 years (n = 1717); (2) presence of stroke at baseline (n = 612), missing stroke information (n = 187), or receiving stroke treatment at baseline (n = 2); (3) age below 45 years (n = 342); (4) missing baseline HDL-C or TC measurements (n = 4575); and (5) outlier values of the TC/HDL-C ratio (three standard deviations above or below the mean) (n = 89). Consequently, our final analysis included a total of 10,184 participants. The study's design and participant flow are shown in Fig. 1.

### Variables

#### TC/HDL-C ratio

Serum TC level (mg/dL) divided by HDL-C level (mg/dL) was used to compute the TC/HDL-C ratio.

### Diagnosis of stroke

The outcome variable in this study was a stroke event during the follow-up period. As previously mentioned [11], data on incident stroke were collected using the following standardized set of inquiries: (1) Were you informed of a stroke diagnosis by a medical professional? (2) When did you initially receive or become aware of the diagnosis? (3) Are you currently undergoing any follow-up treatment related to your stroke? If an individual responded affirmatively to these queries during follow-up, they were classified as having received their first stroke diagnosis, and the self-reported time was documented as the onset of the stroke. The timing of the stroke event was calculated by subtracting the baseline survey time from the stroke onset time. For participants who did not experience a stroke during any follow-up visits, the event onset time was determined by subtracting the baseline survey time from the time of the last survey.

### Covariates

According to previous research findings and clinical expertise, covariates were chosen for this study [11–14]. The following factors were incorporated as covariates: (1) Categorical variables: gender, chronic lung disease (CLD), drinking status, educational attainment, annual income, smoking status, coronary heart disease (CHD), liver disease, physical activity, lipid-lowering medication, antihypertensive medication, and glucose-lowering medication; (2) Continuous variables: age, systolic blood pressure (SBP), diastolic blood pressure (DBP), BMI, C-reactive protein (CRP), white blood cell (WBC), fasting plasma glucose (FPG), hemoglobin, serum creatinine (Scr), Cystatin C, and glycosylated hemoglobin (HbA1c).

### Data collection

The personnel at the CHARLS based at Peking University employed a comprehensive data collection methodology [11]. They instructed the interviewers to utilize the computer-assisted personal interview technique for conducting in-home interviews with the study respondents. The questionnaire administered during these interviews covered various aspects, such as health conditions, demographics, functional status, lifestyle, chronic illnesses diagnosed by a medical professional, and health-related behaviors [11].

Furthermore, to assess the study participants’ health status and physical functioning, the interviewers were equipped with the necessary tools to measure key biometric parameters, including weight, height, and blood pressure. Subjects were invited to nearby Centers for Disease Control and Prevention facilities or their local township hospital as part of the data collection process. Skilled nurses collected an 8 ml fasting blood sample in these healthcare settings. Subsequently, a complete blood count test was conducted within 1-2 hours post-sample collection. Notably, all blood samples were diligently preserved at a local laboratory, maintained at a temperature of 4 °C, and were later transported to the China Center for Disease Control in Beijing within two weeks, with stringent temperature control measures ensuring that a temperature of − 80 °C was consistently maintained throughout the transportation process.

The study defined CHD to encompass a range of cardiovascular ailments, including congestive heart failure, coronary heart disease, myocardial infarction, and other heart diseases [11]. CLD was defined to include conditions such as chronic bronchitis and emphysema [11]. Hypertension was defined as having a blood pressure reading exceeding 140/90 mmHg (based on the average of three measurements) or having a documented history of hypertension [11]. Liver disease was defined to exclude diseases of the liver other than the fatty liver, liver tumors, and liver cancer. Physical activity is defined as at least 1.25 h/week of vigorous activity, 2.5 h/week of moderate-intensity activity, or a combination of both (at least 600 metabolic equivalent minutes/week) [15]. Smoking status was categorized into three distinct groups according to individuals' smoking behavior: current smokers, individuals who have smoked in the past, and individuals who have never smoked. Similarly, drinking status was classified into three categories based on individuals’ drinking behavior: current drinkers, individuals who have previously consumed alcohol, and individuals who have never consumed alcohol.

### Missing data processing

The study’s small fraction of the dataset contained missing values for various clinical variables. Specifically, there were missing data for the following variables: gender (8 individuals, 0.08%), educational attainment (7 individuals, 0.69%), annual income (6166 individuals, 60.55%), drinking status (11 individuals, 0.11%), BMI (1498 individuals, 14.74%), SBP (1959 individuals, 19.24%), DBP (1959 individuals, 19.24%), smoking status (169 individuals, 1.66%), liver disease (64 individuals, 0.63%), physical activity (6007 individuals, 59.08%), CHD (39 individuals, 0.38%), CLD (33 individuals, 0.32%), Scr (5 individuals, 0.05%), Cystatin C (2466 individuals, 24.22%), hemoglobin (233 individuals, 2.29%), WBC (238 individuals, 2.34%), FPG (12 individuals, 0.12%), HbA1c (80 individuals, 0.79%). To address these missing values and ensure the robustness of the analysis, a statistically rigorous approach was employed. The missing clinical variables were imputed using chained equations, and multiple imputations were carried out for modeling purposes [16, 17]. The imputation model incorporated the following variables: age, gender, educational attainment, annual income, smoking status, drinking status, physical activity, BMI, CHD, CLD, liver disease, CRP, WBC, Scr, Cystatin C, hemoglobin, FPG, HbA1c, lipid-lowering medication, antihypertensive medication, and glucose-lowering medication. The analysis of missing data followed the assumption of missing-at-random to ensure the validity of the imputation process [17].

### Statistical analysis

The R statistical software and Empower Stats were used for statistical analyses. The baseline data was initially divided into four groups based on quartiles of the TC/HDL-C ratio. Categorical data were expressed in frequencies and percentages, while continuous data were presented as medians with quartile ranges (25th–75th percentile) or as means with standard deviations (SD). To examine differences among the four groups, we utilized the Kruskal–Wallis H test for data with a skewed distribution, the One-Way ANOVA test for normally distributed data, or the χ^2^ test for categorical data.

We employed multivariate Cox proportional hazards regression models, comprising three distinct models: (1) Model 1: this model did not incorporate any covariates; (2) Model 2: this model adjusted for socio-demographic factors, including age, gender, educational attainment, annual income, smoking status, drinking status, physical activity, and BMI; (3) Model 3: this model adjusted for all factors, including age, gender, educational attainment, annual income, smoking status, drinking status, physical activity, BMI, CHD, CLD, liver disease, CRP, WBC, Scr, Cystatin C, hemoglobin, FPG, HbA1c, lipid-lowering medication, antihypertensive medication, and glucose-lowering medication. To calculate adjusted and unadjusted hazard ratios (HR), we used a 95% confidence interval (CI). We adjusted for that covariate when the HR changed by at least 10% after including a covariate in the model [18]. In a more standard Cox model that predicted only stroke, 162 individuals would be censored from the analysis at the time of death. Information about the risk of death competition was ignored. Competitive risk regression models take into account information from competitive risks and reweight stroke composite endpoint risks based on competitive outcomes. Therefore, competing risks multivariate Cox’s regression was performed with mortality as the competing risk for stroke composite endpoint events. The results were expressed as a subdistribution HR (SHR) with 95% CI.

To validate our findings, we conducted multiple sensitivity analyses. To further assess potential nonlinear patterns, we transformed the continuous variable TC/HDL-C ratio into a ranking variable by the TC/HDL-C ratio quartiles. Then, we calculated P for the trend to validate the TC/HDL-C ratio as a continuous variable. In addition, we conducted sensitivity analyses by excluding individuals with hypertension or CHD to investigate the connection between the TC/HDL-C ratio and stroke in this subgroup. We also calculated E-values to examine the possibility of unmeasured confounding between TC/HDL-C ratio and stroke risk [19].

We employed the Cox proportional hazards regression model with cubic spline functions and smooth curve fitting to assess the non-linear correlation between the TC/HDL-C ratio and incident stroke. If a non-linear correlation was observed, we estimated the inflection point using recursive methods and fitted a two-piecewise Cox proportional hazards regression model for both segments of the inflection point. Ultimately, the selection of the most optimal model for elucidating the connection between the TC/HDL-C ratio and stroke was based on a log-likelihood ratio test.

The study also applied the Cox proportional hazard model to analyze various subgroups, including age, gender, BMI, drinking status, smoking status, physical activity, liver disease, and CLD. BMI (< 25, ≥ 25 kg/m^2^) and age (< 60, ≥ 60 years) were categorized according to clinical cutoffs. Each subgroup analysis underwent comprehensive adjustment, with the exception of the stratification variable. Interactions between subgroups were confirmed using a likelihood ratio test.

The study adhered to the STROBE statement for reporting observational research [18]. Statistical significance was established using a two-tailed test with a threshold of P < 0.05.

## Results

### Characteristics of individuals

This investigation included a cohort of 10,184 individuals without any history of stroke at baseline. The average age of the cohort was 59.16 ± 9.35 years, and throughout a median follow-up duration of 7.0 years, 1192 individuals (11.70%) experienced an incident stroke.

Table 1 comprehensively outlines the baseline characteristics of the study population. The TC/HDL-C ratio was categorized into four groups based on quartiles (Q1 ≤ 3.07; 3.07 < Q2 ≤ 3.80; 3.80 < Q3 ≤ 4.75; Q4 > 4.75). In the Q1 group, participants exhibited elevated levels of age, HDL-C, and Cystatin C, and concurrently lower levels of BMI, SBP, DBP, TG, TC, LDL-C, WBC, CRP, Scr, hemoglobin, FPG, and HbA1c. Conversely, participants in the Q4 group demonstrated lower rates of current drinkers, current smokers, and instances of CLD, alongside heightened rates of physical activity, hyperlipidemia, and CHD.

**Table 1:** The baseline characteristics of participants

TC/HDL-C ratio	Q1 (≤ 3.07)	Q2 (3.07 to ≤ 3.80)	Q3 (3.80 to ≤ 4.75)	Q4 (> 4.75)	P-value
Participants	2546	2546	2546	2546	
Gender					< 0.001
Male	1308 (51.37%)	1138 (44.70%)	1151 (45.21%)	1157 (45.44%)	
Female	1238 (48.63%)	1408 (55.30%)	1395 (54.79%)	1389 (54.56%)	
Age (years)	59.66 ± 9.72	59.30 ± 9.67	58.72 ± 8.90	58.97 ± 9.05	0.002
Educational attainment					0.012
Primary school and below	1845 (72.47%)	1754 (68.89%)	1727 (67.83%)	1737 (68.22%)	
Middle school	456 (17.91%)	520 (20.42%)	539 (21.17%)	533 (20.93%)	
High school and above	245 (9.62%)	272 (10.68%)	280 (11.00%)	276 (10.84%)	
Annual income					0.482
< 10,000 RMB	1582 (62.14%)	1542 (60.57%)	1527 (59.98%)	1510 (59.31%)	
10,000–30000 RMB	577 (22.66%)	598 (23.49%)	615 (24.16%)	636 (24.98%)	
> 30,000 RMB	387 (15.20%)	406 (15.95%)	404 (15.87%)	400 (15.71%)	
BMI (kg/m^2^)	21.98 ± 3.41	23.00 ± 3.89	24.09 ± 3.85	25.15 ± 3.92	< 0.001
SBP (mmHg)	128.05 ± 21.24	129.40 ± 21.72	131.12 ± 21.45	134.45 ± 21.68	< 0.001
DBP (mmHg)	73.79 ± 12.09	74.85 ± 12.02	76.57 ± 12.01	78.22 ± 11.95	< 0.001
Drinking status					< 0.001
Never drinkers	328 (12.88%)	353 (13.86%)	335 (13.16%)	369 (14.49%)	
Ever drinkers	1393 (54.71%)	1558 (61.19%)	1640 (64.41%)	1635 (64.22%)	
Current drinkers	825 (32.40%)	635 (24.94%)	571 (22.43%)	542 (21.29%)	
Smoking status					< 0.001
Never smokers	1474 (57.89%)	1605 (63.04%)	1599 (62.80%)	1592 (62.53%)	
Ever smokers	199 (7.82%)	201 (7.89%)	198 (7.78%)	263 (10.33%)	
Current smokers	873 (34.29%)	740 (29.07%)	749 (29.42%)	691 (27.14%)	
Physical activity					< 0.001
No	1750 (68.74%)	1684 (66.14%)	1619 (63.59%)	1466 (57.58%)	
Yes	796 (31.26%)	862 (33.86%)	927 (36.41%)	1080 (42.42%)	
Liver disease					0.546
No	2438 (95.76%)	2432 (95.52%)	2446 (96.07%)	2451 (96.27%)	
Yes	108 (4.24%)	114 (4.48%)	100 (3.93%)	95 (3.73%)	
CHD					< 0.001
No	2274 (89.32%)	2249 (88.33%)	2251 (88.41%)	2181 (85.66%)	
Yes	272 (10.68%)	297 (11.67%)	295 (11.59%)	365 (14.34%)	
CLD					< 0.001
No	2229 (87.55%)	2284 (89.71%)	2293 (90.06%)	2319 (91.08%)	
Yes	317 (12.45%)	262 (10.29%)	253 (9.94%)	227 (8.92%)	
Hyperlipidaemia					< 0.001
No	2426 (95.29%)	2386 (93.72%)	2313 (90.85%)	2155 (84.64%)	
Yes	120 (4.71%)	160 (6.28%)	233 (9.15%)	391 (15.36%)	
Lipid-lowering medication					0.115
No	2442 (95.92%)	2423 (95.17%)	2426 (95.29%)	2405 (94.46%)	
Yes	104 (4.08%)	123 (4.83%)	120 (4.71%)	141 (5.54%)	
Antihypertensive medication					0.074
No	2083 (81.81%)	2052 (80.60%)	2093 (82.21%)	2027 (79.62%)	
Yes	463 (18.19%)	494 (19.40%)	453 (17.79%)	519 (20.38%)	
Glucose-lowering medication					0.228
No	2464 (96.78%)	2448 (96.15%)	2452 (96.31%)	2436 (95.68%)	
Yes	82 (3.22%)	98 (3.85%)	94 (3.69%)	110 (4.32%)	
TG (mg/dl)	78.24 ± 33.98	100.88 ± 44.23	129.35 ± 56.90	207.72 ± 115.51	< 0.001
LDL-C (mg/dL)	94.50 ± 23.76	113.84 ± 26.07	125.26 ± 29.50	132.29 ± 42.34	< 0.001
TC (mg/dl)	174.00 ± 32.22	186.12 ± 32.58	196.55 ± 33.39	214.20 ± 40.12	< 0.001
HDL-C (mg/dL)	67.22 ± 14.76	54.42 ± 9.86	46.56 ± 8.27	37.36 ± 7.69	< 0.001
WBC (× 10^9^)					
CRP (mg/l)	0.75 (0.44–1.68)	0.88 (0.49–1.87)	1.07 (0.60–2.18)	1.38 (0.77–2.73)	< 0.001
Scr (mg/dl)	0.77 ± 0.34	0.77 ± 0.19	0.78 ± 0.18	0.81 ± 0.20	< 0.001
Cystatin C (mg/L)	1.03 ± 0.31	1.02 ± 0.27	1.00 ± 0.23	0.96 ± 0.28	< 0.001
Hemoglobin (g/L)	14.08 ± 2.22	14.25 ± 2.16	14.60 ± 2.30	14.78 ± 2.20	< 0.001
FPG (mg/dl)	103.00 ± 26.06	105.44 ± 26.28	109.54 ± 36.05	120.21 ± 45.33	< 0.001
HbA1c (%)	5.10 ± 0.60	5.17 ± 0.64	5.26 ± 0.77	5.44 ± 1.06	< 0.001

Values are n (%) or mean ± SD or median (quartile)

*TC/HDL-C ratio* total cholesterol to high-density lipoprotein cholesterol ratio, *BMI* body mass index, *SBP* systolic blood pressure, *DBP* diastolic blood pressure, *CLD* chronic lung diseases, *CHD* coronary heart disease, *TG* triglycerides, *LDL-C* low-density lipoprotein cholesterol, *TC* total cholesterol, *HDL-C* high-density lipoprotein cholesterol, *WBC* White blood cell, *CRP* C-reactive protein, *Scr* serum creatinine, *FPG* fasting plasma glucose, *HbA1c* glycosylated hemoglobin

#### The incidence rate of stroke

Table 2 presents the stroke incidence rates observed in a cohort of 10,184 individuals during the follow-up period. In the male cohort, the incidence rates of stroke varied significantly with age. For participants under 60 years of age, the incidence rate was 8.71% (95%CI 7.60%–9.81%), translating to 13.69 events per 1000 person-years. In the 60–70 age group, the incidence rate increased to 12.47% (95% CI 10.77%–14.18%), or 20.79 events per 1000 person-years. The 70–80 age group saw a further increase in incidence rate to 14.85% (95% CI 12.18%–17.51%), equivalent to 28.17 events per 1000 person-years. The incidence rate for males aged 80 and above was slightly lower at 12.50% (95% CI 6.28%–18.72%), with 27.59 events per 1000 person-years. In the female cohort, the pattern of increasing stroke incidence rates with age was also observed. Females under 60 years of age had an incidence rate of 10.40% (95% CI 9.34%–11.47%), or 16.41 events per 1000 person-years. The incidence rate for the 60–70 age group was 14.55% (95% CI 12.76%–16.35%), translating to 23.74 events per 1000 person-years. The 70–80 age group experienced the highest incidence rate among females, at 18.04% (95% CI 15.00%–21.07%), or 32.44 events per 1000 person-years. Females aged 80 and above had an incidence rate of 12.33% (95% CI 6.93%–17.73%), with 26.46 events per 1000 person-years.

**Table 2:** Incidence rate of incident stroke

	Participants (n)	Stroke events (n)	Incidence rate (95%CI) (%)	Per 1000 person-year
Total	10,184	1192	11.70 (11.08–12.33)	19.19
Male				
Age (years)				
< 60	2504	218	8.71 (7.60–9.81)	13.69
60–70	1451	181	12.47 (10.77–14.18)	20.79
70–80	687	102	14.85 (12.18–17.51)	28.17
≥ 80	112	14	12.50 (6.28–18.72)	27.59
Female				
Age (years)				
< 60	3172	330	10.40 (9.34–11.47)	16.41
60–70	1491	217	14.55 (12.76–16.35)	23.74
70–80	621	112	18.04 (15.00–21,07)	32.44
≥ 80	146	18	12.33 (6.93–17.73)	26.46

*CI* confidence interval

### The results of the correlation between the TC/HDL-C ratio and incident stroke

As the TC/HDL-C ratio met the proportional hazards assumption, the association between the TC/HDL-C ratio and stroke risk was evaluated by the Cox proportional hazards regression model. Table 3 presents the outcomes of the Cox proportional hazard regression model, assessing the relationship between the TC/HDL-C ratio and the occurrence of incident stroke. In Model 1, where no covariates were considered, the TC/HDL-C ratio exhibited a positive association with incident stroke (HR: 1.14, 95%CI 1.09–1.19, P < 0.0001). After controlling for covariates including age, gender, educational attainment, annual income, smoking status, drinking status, physical activity, and BMI in Model 2, an 8% escalation in the risk of stroke associated with each incremental unit rise in the TC/HDL-C ratio (HR = 1.08, 95%CI 1.04–1.13). In Model 3, after adjusting for age, gender, educational attainment, annual income, smoking status, drinking status, physical activity, BMI, CHD, CLD, liver disease, CRP, WBC, Scr, Cystatin C, hemoglobin, FPG, HbA1c, lipid-lowering medication, antihypertensive medication, and glucose-lowering medication, the HR (95%CI) was 1.05 (1.00–1.10). This revealed that for each unit increase in the TC/HDL-C ratio, the stroke risk increased by 5%.

**Table 3:** Relationship between TC/HDL-C ratio and the incident stroke in different models

Exposure	Model 1 (HR.,95% CI, P)	Model 2 (HR,95% CI, P)	Model 3 (HR,95% CI, P)
TC/HDL-C ratio (per 1 increase)	1.14 (1.09, 1.19) < 0.0001	1.08 (1.04, 1.13) 0.0003	1.05 (1.00, 1.10) 0.0402
TC/HDL-C ratio (quartile)			
Q1	Ref	Ref	Ref
Q2	1.28 (1.08, 1.53) 0.0050	1.21 (1.01, 1.44) 0.0360	1.21 (1.02, 1.44) 0.0319
Q3	1.38 (1.17, 1.64) 0.0002	1.24 (1.04, 1.47) 0.0161	1.25 (1.04, 1.48) 0.0145
Q4	1.63 (1.38, 1.93) < 0.0001	1.36 (1.14, 1.61) 0.0005	1.23 (1.02, 1.47) 0.0258
P for trend	< 0.0001	0.0008	0.0384

Model 1: we did not adjust for other covariants

Model 2: we adjusted for age, gender, educational attainment, annual income, smoking status, drinking status, physical activity, and BMI

Model 3: we adjusted for age, gender, educational attainment, annual income, smoking status, drinking status, physical activity, BMI, CHD, CLD, liver disease, CRP, WBC, Scr, Cystatin C, hemoglobin, FPG, HbA1c, lipid-lowering medication, antihypertensive medication, and glucose-lowering medication

*HR* hazard ratios, *CI* confidence interval, *Ref* reference, *TC/HDL-C ratio* total cholesterol to high-density lipoprotein cholesterol ratio

### The results of competing risks multivariate Cox’s regression

When death was treated as a competing event, the competing analysis results were shown in Additional file 1: Table S1. In the crude model, the TC/HDL-C ratio was positively associated with the stroke composite endpoint (SHR = 1.14, 95%CI 1.09–1.19). The minimally adjusted model (Model I) (adjusted age, gender, educational attainment, annual income, smoking status, drinking status, physical activity, and BMI) did not show the apparent change (SHR: 1.08, 95%CI 1.04–1.13). In the fully adjusted model (Model II) (adjusted age, gender, educational attainment, annual income, smoking status, drinking status, physical activity, BMI, CHD, CLD, liver disease, CRP, WBC, Scr, Cystatin C, hemoglobin, FPG, HbA1c, lipid-lowering medication, antihypertensive medication, and glucose-lowering medication), we could also detect the connection (SHR = 1.05, 95%CI 1.01–1.10). This result was similar to when the competing risk of death was not considered.

### Sensitivity analyses

In order to fortify the reliability of our findings, a comprehensive set of sensitivity analyses was systematically conducted. Initially, the TC/HDL-C ratio underwent a transformation from a continuous variable to a categorical variable. Subsequently, this categorically transformed ratio was reintroduced into the model. Post-transformation, the results indicated non-equidistant trends in effect sizes among groups, suggesting a potential non-linear association between the TC/HDL-C ratio and incident stroke.

Additionally, we conducted sensitivity analyses focusing on individuals without hypertension. Following the adjustment for confounding covariates, a positive relationship between the TC/HDL-C ratio and stroke risk persisted (HR = 1.07, 95% CI 1.00–1.13, P = 0.0433) (Table 4, Model 4). Furthermore, we extended our analyses to individuals without CHD. The outcomes demonstrated a sustained positive linkage between the TC/HDL-C ratio and stroke risk, even after comprehensive adjustment for a multitude of covariates (HR = 1.09, 95% CI 1.03–1.15, P = 0.0036) (Table 4, Model 5). Moreover, an E-value was computed to assess the vulnerability of the study results to potential unobserved confounding factors. The resulting E-value (1.28) demonstrated a higher level of statistical significance in comparison to the relative risk (1.26) associated with unmeasured confounders and TC/HDL-C ratio. This suggests that the impact of unmeasured or unidentified confounders on the relationship between TC/HDL-C ratio and stroke risk was negligible. These additional sensitivity analyses underscored the robustness of our results.

**Table 4:** Relationship between TC/HDL-C ratio and stroke risk in different sensitivity analyses

Exposure	Model 4 (HR, 95%CI, P)	Model 5 (HR, 95%CI, P)
TC/HDL-C ratio (per 1 increase)	1.07 (1.00, 1.13) 0.0433	1.09 (1.03, 1.15) 0.0036
TC/HDL-C ratio (quartile)		
Q1	Ref	Ref
Q2	1.26 (1.01, 1.57) 0.0446	1.28 (1.02, 1.61) 0.0328
Q3	1.23 (0.98, 1.55) 0.0695	1.35 (1.07, 1.69) 0.0107
Q4	1.28 (1.01, 1.63) 0.0399	1.45 (1.15, 1.83) 0.0015
P for trend	0.0582	0.0023

Model 4 was sensitivity analysis in participants without hypertension. We adjusted for age, gender, educational attainment, annual income, smoking status, drinking status, physical activity, BMI, CHD, CLD, Liver disease, CRP, WBC, Scr, Cystatin C, hemoglobin, FPG, HbA1c, lipid-lowering medication, antihypertensive medication, and glucose-lowering medication

Model 5 was sensitivity analysis in participants without CHD. We adjusted for age, gender, educational attainment, annual income, smoking status, drinking status, physical activity, BMI, CLD, liver disease, CRP, WBC, Scr, Cystatin C, hemoglobin, FPG, HbA1c, lipid-lowering medication, antihypertensive medication, and glucose-lowering medication

*HR* hazard ratios, *CI* confidence, *Ref* reference, *TC/HDL-C ratio* total cholesterol to high-density lipoprotein cholesterol ratio

### The analysis of the non-linear correlation

**Fig. 2:**
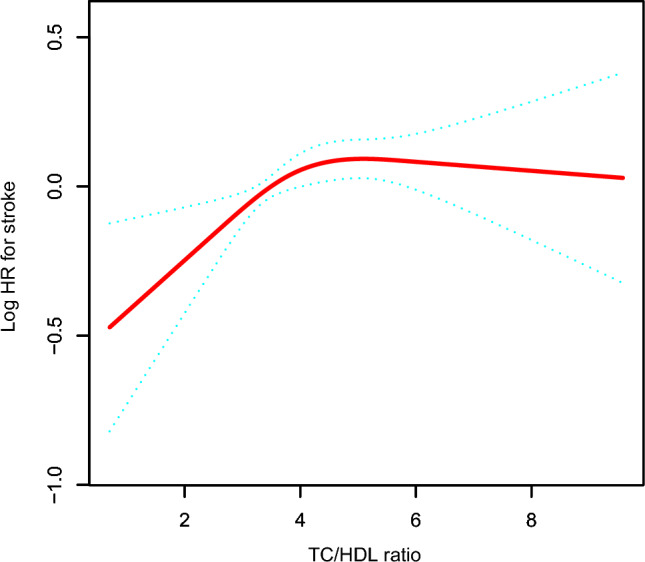
The non-linear relationship between TC/HDL-C ratio and incident stroke. A non-linear relationship between TC/HDL-C ratio and stroke was detected after adjustment for age, gender, educational attainment, annual income, smoking status, drinking status, physical activity, BMI, CLD, liver disease, CRP, WBC, Scr, Cystatin C, hemoglobin, FPG, HbA1c, lipid-lowering medication, antihypertensive medication, and glucose-lowering medication

Figure 2 displays the non-linear correlation between the TC/HDL-C ratio and the incidence of stroke. Following adjustments for various covariates, including age, gender, educational attainment, annual income, smoking status, drinking status, physical activity, BMI, CHD, CLD, liver disease, CRP, WBC, Scr, Cystatin C, hemoglobin, FPG, HbA1c, lipid-lowering medication, antihypertensive medication, and glucose-lowering medication, a non-linear correlation between the TC/HDL-C ratio and the risk of stroke was identified (Table 5). The current investigation revealed an inflection point in the TC/HDL-C ratio at 3.71, as determined by a two-piecewise Cox proportional hazards regression model (P for log-likelihood ratio test = 0.016). Below this inflection point, a positive correlation was observed between the TC/HDL-C ratio and the probability of diabetes (HR: 1.25, 95%CI 1.07–1.45, P = 0.0039). Conversely, when the TC/HDL-C ratio exceeded 3.71, this correlation was not statistically significant (HR: 1.00, 95% CI 0.94–1.06, P = 0.9232).

**Table 5:** The result of the two-piecewise Cox proportional hazards regression model

Incident stroke	HR (95%CI)	P
Fitting model by standard linear regression (per 1 increase in TC/HDL-C ratio)	1.05 (1.00, 1.10)	0.0410
Fitting model by two-piecewise Cox proportional hazards		
The inflection point of the TC/HDL-C ratio	3.71	
≤ 3.71 (per 1 increase in TC/HDL-C ratio)	1.25 (1.07, 1.45)	0.0039
> 3.71 (per 1 increase in TC/HDL-C ratio)	1.00 (0.94, 1.06)	0.9232
P for the log-likelihood ratio test	0.016	

We adjusted for age, gender, educational attainment, annual income, smoking status, drinking status, physical activity, BMI, CLD, liver disease, CRP, WBC, Scr, Cystatin C, hemoglobin, FPG, HbA1c, lipid-lowering medication, antihypertensive medication, and glucose-lowering medication

*HR* hazard ratios, *CI* confidence, *TC/HDL-C ratio* total cholesterol to high-density lipoprotein cholesterol ratio

### The results of the subgroup analysis

Subgroup analysis was employed as a methodological approach to discern potential confounding variables that might have exerted an influence on the association between the TC/HDL-C ratio and the occurrence of incident stroke (Table 6). Stratification was conducted based on pertinent variables: BMI, gender, age, drinking status, physical activity, smoking status, liver disease, CHD, and CLD. It is noteworthy that after rigorous examination, all of the aforementioned covariates were found to exert no discernible impact on the nexus between the TC/HDL-C ratio and the occurrence of incident stroke.

**Table 6:** Effect size of TC/HDL-C ratio on stroke in prespecified and exploratory subgroups

Characteristic	No of participants	HR (95%CI) P value	P for interaction
Age (years)			0.5714
< 60	5676	1.06 (1.00, 1.13) 0.0553	
≥ 60	4508	1.04 (0.98, 1.10) 0.2399	
Gender			0.6418
Male	4754	1.07 (1.00, 1.14) 0.0507	
Female	5430	1.05 (0.98, 1.11) 0.1442	
BMI (kg/m^2^)			0.2616
< 25	6921	1.08 (1.02, 1.15) 0.0086	
≥ 25	3263	1.03 (0.97, 1.10) 0.3598	
Liver disease			0.1123
No	9767	1.05 (1.00, 1.10) 0.0515	
Yes	417	1.20 (1.02, 1.41) 0.0235	
CLD			0.2862
No	9125	0.98 (0.87, 1.12) 0.8169	
Yes	1059	1.06 (1.01, 1.11) 0.0171	
Smoking status			0.1261
Never smokers	6270	1.07 (1.01, 1.13) 0.0215	
Ever smokers	861	1.18 (1.03, 1.35) 0.0138	
Current smokers	3053	0.97 (0.89, 1.06) 0.5454	
Drinking status			0.2178
Never drinkers	1385	1.05 (0.94, 1.18) 0.3885	
Ever drinkers	6226	1.10 (1.04, 1.16) 0.0016	
Current drinkers	2573	0.97 (0.88, 1.07) 0.5691	
Physical activity			0.2456
No	3665	1.08 (1.02, 1.15) 0.0124	
Yes	6519	1.03 (0.97, 1.10) 0.3322	

The above model was adjusted for age, gender, educational attainment, annual income, smoking status, drinking status, physical activity, BMI, CLD, liver disease, CRP, WBC, Scr, Cystatin C, hemoglobin, FPG, HbA1c, lipid-lowering medication, antihypertensive medication, and glucose-lowering medication

The model is not adjusted for the stratification variable in each case

## Discussion

In this cohort study, an investigation was conducted to examine the association between the TC/HDL-C ratio and stroke risk in a population of middle-aged and older subjects in China. The findings of this research demonstrated a significant positive connection between the TC/HDL-C ratio and the occurrence of stroke. Moreover, an inflection point was identified, and different connections between the TC/HDL-C ratio and stroke risk were observed on either side of this point. Furthermore, the reliability of our results was confirmed through sensitivity analyses and subgroup analyses.

In recent years, there has been a notable increase in the prevalence of stroke within the general Chinese population, reaching a rate of 2.47 per 1000 person-years [20]. However, the present study reveals a significantly higher incidence of stroke, with a rate of 19.19 per 1000 person-years. This discrepancy may be attributed to the fact that the study population consisted exclusively of individuals aged 45 and above. Consequently, it is reasonable to expect a substantially elevated incidence of stroke among the study participants compared to the general population. Acknowledging that this cohort exhibits a considerably higher stroke incidence rate than the overall population is crucial. Thus, it is crucial to assess potential stroke risk factors in middle-aged and older adults.

Dyslipidemia plays a pivotal role in the pathogenesis of stroke [21], with TC, LDL-C, and TG being routinely assessed in clinical settings. Nevertheless, the limited predictive capacity of single lipid indices for cardiovascular diseases has prompted an exploration of non-traditional lipid profiles [21], notably the TC/HDL-C ratio, which has garnered increasing attention. Despite established evidence linking an elevated TC/HDL-C ratio to heightened cardiovascular disease risk, its association with stroke risk remains a subject of ongoing debate. In a prospective cohort study involving 5099 hypertensive individuals, spanning a median follow-up of 8.4 years, each one-unit increase in the TC/HDL-C ratio demonstrated an 18% escalation in the risk of ischemic stroke after adjustments for BMI, age, DBP, ethnicity, heavy drinking, sex, SBP, diabetes mellitus, current smoking, and antihypertensive medications (HR:1.18, 95%: 1.06–1.31) [5]. Furthermore, a prospective cohort study involving 34,294 participants corroborated the link between an augmented TC/HDL-C ratio and an increased stroke risk, even following adjustments for an extensive array of covariates, including retirement status, sex, age, education level, alcohol consumption, BMI, physical activities, smoking index, chronic kidney disease, hypertension, diabetes, and hyperuricemia [6]. However, discordant findings exist within the literature. A prospective study encompassing 58,235 Finnish individuals without baseline coronary heart disease or stroke failed to establish a statistically significant connection between the TC/HDL-C ratio and the risk of ischemic stroke, subarachnoid hemorrhagic stroke, or intracerebral hemorrhagic stroke after adjusting for relevant risk factors [8]. Similarly, another prospective cohort study involving 24,566 participants without cardiovascular disease found no connection between TC/HDL-C ratios and stroke risk, even after adjusting for current smoking, age, BMI, uric acid, systolic blood pressure, and HbA1c [9]. The incongruities in these findings may be attributed to variations in the range of TC/HDL-C ratios, sample sizes, and the diverse set of adjustment variables employed across studies. Additionally, the potential for non-linear relationships, which may alter the conclusions drawn from linear regression analyses, could contribute to the observed disparities. Our results supported the literature's claim that an increased TC/HDL-C ratio increases new-onset stroke risk. Notably, sensitivity analyses revealed a persistent connection between an elevated TC/HDL-C ratio and incident stroke among individuals without hypertension and coronary heart disease. These insights contribute to our understanding of stroke risk in middle-aged and elderly adults, facilitating informed strategies for risk reduction in this demographic.

The precise connection between the TC/HDL-C ratio and stroke remains uncertain. We speculated that the connection between TC/HDL-C ratio and stroke may be due to the following mechanisms. Firstly, elevated TC or decreased HDL-C are intricately linked to an augmented susceptibility to atherosclerosis, characterized by the accretion of lipid plaques within arterial walls, culminating in the constriction and rigidification of blood vessels [23, 24]. Secondly, an elevated TC/HDL-C ratio is related to heightened levels of inflammation and oxidative stress, both of which are implicated in the initiation and progression of atherosclerosis [24–27]. Furthermore, an increased TC/HDL-C ratio is related to endothelial dysfunction, wherein the impairment of the inner lining of blood vessels compromises the regulation of blood vessel tone, thereby contributing to atherosclerosis and clot formation [24, 28, 29].

Moreover, the present investigation has illuminated a non-linear association between the TC/HDL-C ratio and stroke. Employing a two-piecewise Cox proportional hazards regression model, this study endeavored to elucidate the intricacies of the non-linear correlations observed comprehensively. The identified connection between the TC/HDL-C ratio and stroke among individuals in the middle-aged and elderly demographic exhibited a non-linear trajectory characterized by a saturation effect. Following adjustment for confounding variables, the inflection point of the TC/HDL-C ratio was determined to be 3.71. Notably, below this threshold, the risk of stroke escalated by 25% for each incremental unit rise in the TC/HDL-C ratio (HR: 1.25, 95%CI 1.07–1.45). However, beyond the inflection point of 3.71, elevating the TC/HDL-C ratio did not contribute to a concomitant increase in the likelihood of stroke (HR: 1.00, 95% CI 0.94–1.06). The findings of this study provide a robust theoretical underpinning for advocating the reduction of the TC/HDL-C ratio in clinical settings, particularly when the ratio falls below the identified inflection point of 3.71. Additionally, the insights gleaned from this research offer valuable information to guide medical practitioners in assisting patients with varying TC/HDL-C ratios in mitigating the risk of stroke.

The present study elucidates several noteworthy strengths. Firstly, it stands as the inaugural investigation, to the best of our knowledge, probing the nexus between the TC/HDL-C ratio and incident stroke specifically within the middle-aged and elderly demographic. Secondly, the research boasts a commendably extensive sample size, augmenting the statistical robustness of our findings. Thirdly, a distinctive facet of our inquiry is meticulously examining the non-linear connection between the TC/HDL-C ratio and stroke. Fourthly, the study demonstrates methodological rigor through the minimization of residual confounding factors. The implementation of stringent statistical adjustments serves to enhance the internal validity of our results. Moreover, the sensitivity analyses, wherein the TC/HDL-C ratio was transformed into a categorical variable, and associations reevaluated after excluding individuals with hypertension and CHD, further corroborate the robustness of our findings. Fifthly, the research adopts a thorough subgroup analysis, scrutinizing potential confounding variables that might exert an influence on the connection between the TC/HDL-C ratio and stroke.

Despite these strengths, it is imperative to acknowledge certain limitations inherent in the current investigation. Firstly, the participant pool exclusively comprised individuals aged 45 and above of Chinese ethnicity, necessitating caution in extrapolating our findings to other ethnicities and younger age groups. Validation studies within diverse demographic cohorts are warranted to ascertain the generalizability of our results. Secondly, it is important to highlight that stroke diagnosis in this study was solely based on self-report, without considering information on stroke-related symptoms (such as TIA) and imaging findings. Although the absence of CHARLS validation data specifically for self-reported stroke diagnosis is acknowledged, previous stroke epidemiological studies have reported sensitivity and specificity rates ranging from 78 to 96% and 96% to 98% [30–32] when using self-report. Future research aims to develop comprehensive studies incorporating information on stroke-related symptoms and imaging findings to enhance the accuracy of stroke diagnosis. Thirdly, the inability to differentiate between various stroke subtypes poses a notable limitation. The ambiguity surrounding the specific associations with hemorrhagic and ischemic strokes precludes a nuanced understanding of the differential impact of the TC/HDL-C ratio on distinct stroke subcategories. Fourthly, unmeasured or uncontrollable confounders, such as inflammation indicators, family history of stroke, liver function indicators, and diet, cannot be entirely ruled out despite meticulous control over recognized potential confounders. Nevertheless, we employed the E-value to assess the influence of unmeasured confounders and found it improbable that they accounted for the outcomes. In the future, we can consider designing our studies with a larger and more diverse sample size to collect as many variables as possible, including information on inflammation indicators, family history of stroke, liver function indicators, and diet.

## Conclusion

Our investigation reveals a discernible, non-linear (inverted L-shaped) dose–response relationship between the TC/HDL-C ratio and the risk of stroke in individuals aged 45 years or older. An upward trajectory in the TC/HDL-C ratio is concomitantly associated with an escalated risk of stroke. Notably, the risk of experiencing a stroke reaches its zenith when the TC/HDL-C ratio surpasses the threshold of 3.71. The implications of our findings imply that the mitigation of stroke risk in clinical settings may be achieved through the targeted reduction of the TC/HDL-C ratio, particularly when it falls below 3.71.

### Supplementary Information

**Additional file 1: Table S1.** Relationship between TC/HDL-C ratio and the stroke composite endpoint in different models with competing risk of mortality. **Table S2.** Relationship between TC/HDL-C ratio and the incident stroke in different models. **Table S3.** Relationship between TC/HDL-C ratio and the incident stroke in different models after excluding participants with missing values for BMI, physical activity, and Cystatin C.

## Data Availability

Data are available from http://www.isss.pku.edu.cn/cfps/. Follow the prompts to register as a user and download the data once it has been reviewed and approved.

## Abbreviations

TC/HDL-C ratio: Triglyceride to high-density lipoprotein cholesterol ratio
CHARLS: China Health and Retirement Longitudinal Study
BMI: Body mass index
FPG: Fasting plasma glucose
TC: Total cholesterol
TG: Triglyceride
HDL-C: High-density lipoprotein cholesterol
LDL-C: Low-density lipid cholesterol
Scr: Serum creatinine
HbA1c: Glycosylated hemoglobin
CRP: C-reactive protein
CLD: Chronic lung disease
CHD: Coronary heart disease
HR: Hazard ratios
CI: Confidence intervals
Ref: Reference
SHR: Subdistribution
