# Supplementary material for: A non-linear connection between the total cholesterol to high-density lipoprotein cholesterol ratio and stroke risk: a retrospective cohort study from the China Health and Retirement Longitudinal Study
Source: Eur J Med Res. 2024 Mar 16;29:175. doi: 10.1186/s40001-024-01769-9 (PMC10943863; doi:10.1186/s40001-024-01769-9)
Supplement: Supplementary file 1 — Additional file 1: Table S1. Relationship between TC/HDL-C ratio and the stroke composite endpoint in different models with competing risk of mortality. Table S2. Relationship between TC/HDL-C ratio and the incident stroke in different models. Table S3. Relationship between TC/HDL-C ratio and the incident stroke in different models after excluding participants with missing values for BMI, physical activity, and Cystatin C. [file 40001_2024_1769_MOESM1_ESM.docx]

**Table S1** Relationship between TC/HDL-C ratio and the stroke composite endpoint in different models with competing risk of mortality

| Exposure | Crude model (SHR,95%CI, P) | Model I (SHR,95%CI, P) | Model II (SHR,95%CI, P) |
| --- | --- | --- | --- |
| TC/HDL-C ratio (per 1 increase) | 1.14 (1.09, 1.19) <0.0001 | 1.08 (1.04, 1.13) 0.0003 | 1.05 (1.01, 1.10) 0.0222 |
| TC/HDL-C ratio (quartile) |  |  |  |
| Q1 | ref | ref | ref |
| Q2 | 1.28 (1.08, 1.53) 0.0050 | 1.21 (1.01, 1.44) 0.0360 | 1.21 (1.02, 1.45) 0.0299 |
| Q3 | 1.38 (1.17, 1.64) 0.0002 | 1.24 (1.04, 1.47) 0.0161 | 1.26 (1.05, 1.50) 0.0108 |
| Q4 | 1.63 (1.38, 1.93) <0.0001 | 1.36 (1.14, 1.61) 0.0005 | 1.25 (1.05, 1.50) 0.0135 |
| P for trend | <0.0001 | 0.0008 | 0.0384 |

Model 1: we did not adjust for other covariants.

Model 2: we adjusted for age, gender, educational attainment, annual income, smoking status, drinking status, physical activity, and BMI.

Model 3: we adjusted for age, gender, educational attainment, annual income, smoking status, drinking status, physical activity, BMI, CHD, CLD, liver disease, CRP, WBC, Scr, Cystatin C, hemoglobin, FPG, HbA1c, lipid-lowering medication, antihypertensive medication, and glucose-lowering medication.

SHR, subdistribution; CI: confidence interval; Ref: reference; TC/HDL-C ratio: total cholesterol to high-density lipoprotein cholesterol ratio

**Table S2** Relationship between TC/HDL-C ratio and the incident stroke in different models

| Exposure | Model 1 (HR.,95% CI, P) | Model 2 (HR,95% CI, P) | Model 3 (HR,95% CI, P) |
| --- | --- | --- | --- |
| TC/HDL-C ratio (per 1 increase) | 1.14 (1.09, 1.19) <0.0001 | 1.11 (1.07, 1.16) <0.0001 | 1.07 (1.02, 1.12) 0.0023 |
| TC/HDL-C ratio (quartile) |  |  |  |
| Q1 | ref | ref | ref |
| Q2 | 1.28 (1.08, 1.53) 0.0050 | 1.24 (1.04, 1.48) 0.0154 | 1.24 (1.04, 1.48) 0.0160 |
| Q3 | 1.38 (1.17, 1.64) 0.0002 | 1.31 (1.11, 1.56) 0.0019 | 1.30 (1.10, 1.55) 0.0027 |
| Q4 | 1.63 (1.38, 1.93) <0.0001 | 1.48 (1.25, 1.75) <0.0001 | 1.32 (1.11, 1.57) 0.0017 |
| P for trend | <0.0001 | <0.0001 | 0.0024 |

Note: we did not adjust for BMI, physical activity, and Cystatin C in Model 1, Model 2, and Model 3.

Model 1: we did not adjust for other covariants.

Model 2: we adjusted for age, gender, educational attainment, annual income, smoking status, and drinking status.

Model 3: we adjusted for age, gender, educational attainment, annual income, smoking status, drinking status, CHD, CLD, liver disease, CRP, WBC, Scr, hemoglobin, FPG, HbA1c, lipid-lowering medication, antihypertensive medication, and glucose-lowering medication.

HR: hazard ratios; CI: confidence interval; Ref: reference; TC/HDL-C ratio: total cholesterol to high-density lipoprotein cholesterol ratio

**Table S3** Relationship between TC/HDL-C ratio and the incident stroke in different models after excluding participants with missing values for BMI, physical activity, and Cystatin C

| Exposure | Model 1 (HR.,95% CI, P) | Model 2 (HR,95% CI, P) | Model 3 (HR,95% CI, P) |
| --- | --- | --- | --- |
| TC/HDL-C ratio (per 1 increase) | 1.12 (1.07, 1.18) <0.0001 | 1.08 (1.03, 1.14) 0.0026 | 1.06 (1.00, 1.11) 0.0491 |
| TC/HDL-C ratio (quartile) |  |  |  |
| Q1 | ref | ref | ref |
| Q2 | 1.31 (1.07, 1.60) 0.0080 | 1.25 (1.02, 1.53) 0.0318 | 1.26 (1.03, 1.54) 0.0275 |
| Q3 | 1.44 (1.19, 1.76) 0.0002 | 1.35 (1.11, 1.65) 0.0031 | 1.38 (1.12, 1.68) 0.0020 |
| Q4 | 1.54 (1.27, 1.88) <0.0001 | 1.36 (1.11, 1.66) 0.0028 | 1.26 (1.02, 1.56) 0.0294 |
| P for trend | <0.0001 | 0.0025 | 0.0258 |

Model 1: we did not adjust for other covariants.

Model 2: we adjusted for age, gender, educational attainment, annual income, smoking status, drinking status, physical activity, and BMI.

Model 3: we adjusted for age, gender, educational attainment, annual income, smoking status, drinking status, physical activity, BMI, CHD, CLD, liver disease, CRP, WBC, Scr, Cystatin C, hemoglobin, FPG, HbA1c, lipid-lowering medication, antihypertensive medication, and glucose-lowering medication.

HR: hazard ratios; CI: confidence interval; Ref: reference; TC/HDL-C ratio: total cholesterol to high-density lipoprotein cholesterol ratio
